# The Extent and Nature of Food and Beverage Company Sponsorship of Children’s Sports Clubs in Canada: A Pilot Study

**DOI:** 10.3390/ijerph17093023

**Published:** 2020-04-27

**Authors:** Elise Pauzé, Odera Ekeh, Monique Potvin Kent

**Affiliations:** 1 School of Epidemiology and Public Health, Faculty of Medicine, University of Ottawa, 600 Peter Morand Crescent, Ottawa, ON K1G 3Z3, Canada; epauz022@uottawa.ca; 2 Department of Biology, Faculty of Science, University of Ottawa, 30 Marie Curie, Ottawa, ON K1N 6N5, Canada; oekeh055@uottawa.ca

**Keywords:** food marketing, children, adolescents, sponsorship, sports, health policy, corporate social responsibility, alcohol

## Abstract

Food and beverage marketing is considered a determinant of childhood obesity. Sponsorship is a marketing technique used by the food industry to target young people when they are engaged in sports. The purpose of this study was to document the frequency and nature of food company sponsorship of children’s sports clubs in Ottawa, Canada. Using national data on sports participation, the five most popular sports among Canadian children aged 4–15 years were first selected for inclusion in the study and relevant sports clubs located in Ottawa (Canada) were then identified. Sports club websites were reviewed between September and December 2018 for evidence of club sponsorship. Food company sponsors were identified and classified by food category. Of the 67 sports clubs identified, 40% received some form of food company sponsorship. Overall, sports clubs had 312 commercial and noncommercial sponsors. Food companies constituted 16% of total sponsors and were the second most frequent type of sponsor after sports-related goods, services, and retailers (25%). Fast food restaurants and other restaurants accounted for 45% and 41% of food company sponsors, respectively. Food company sponsorship of children’s sports clubs is frequent with some promoting companies or brands associated with unhealthy foods. Policymakers should consider restricting the sponsorship of children’s sports clubs by food companies that largely sell or promote unhealthy foods.

## 1. Introduction

Obesity and poor diet increase the risk of developing chronic diseases, including cardiovascular disease, stroke, hypertension, type 2 diabetes, and cancer [1]. Childhood obesity is particularly concerning as the prevalence has reached alarming proportions worldwide [2]. In Canada, nearly one in three children aged 6–17 years have excess weight or obesity with 52–57% of their caloric intake, on average, coming from ultraprocessed foods [3,4].

While there are many contributing factors, the role of unhealthy food marketing in shaping children’s dietary behaviors and nutritional health has garnered increased attention over the last decade and a half. Studies have shown that food marketing is frequently child-directed and most often promotes highly processed and energy dense food products such as fast foods, soft drinks, candy, savory snacks, and sugary breakfast cereals, which are all inconsistent with dietary recommendations [5,6,7,8]. This is concerning as multiple systematic reviews have demonstrated a link between children’s exposure to food marketing and their food preferences, food purchase requests, and their short-term food intake [8,9,10,11,12]. Children’s vulnerability to advertising is underpinned by evidence showing that children under 12 years old do not have the cognitive abilities to fully and consistently understand the persuasive intent of advertising and are thus extremely susceptible to its influence [13,14]. As such, many national and international health organizations, including the World Health Organization, have called on governments to restrict unhealthy food marketing to children [15,16].

The marketing of food by way of sports is considered a particularly persuasive promotional technique [17]. Food company sponsorship is one type of sports-related marketing to which children are exposed [18,19,20,21,22,23,24]. Sponsorship has been defined as “the provision of assistance either financial or in-kind to an activity by a commercial organization for the purpose of achieving commercial objectives” [25]. Food company sponsorship of sports is in fact pervasive and often promotes unhealthy food products or brands [21,22,23,24,26,27,28,29,30,31]. In the context of children’s sports, promotional activities related to sponsorship that may benefit sponsors include naming rights for clubs or teams, branding uniforms, sports equipment, and sporting rewards, signage on sports club premises, and the provision of coupons or discounts to sports club members, among others [21].

Notably, research on tobacco, alcohol, and food company sponsorship has shown that this marketing technique is effective at increasing brand recognition and recall, creating implicit associations between sponsors and sport, as well as cultivating favorable attitudes among consumers, including children and youth, towards sponsoring companies [32,33,34,35,36,37,38]. These cognitive and affective effects likely influence consumption behaviors [39,40]. For instance, the viewership of professional sporting events sponsored by alcohol or tobacco manufacturers has been associated with alcohol consumption and a higher likelihood of smoking among youth, respectively [32,41].

While it is generally known that food companies market to children by sponsoring their sports programs or clubs [18,19], few studies have documented the extent or reach of this practice systematically [20,22]. In Australia, research conducted on 108 child sports clubs in New South Wales found that 17% of club sponsors were food-related and half of these were not deemed “healthy” sponsors [21]. Collectively, it was estimated that Australian children engaged in sports known to be sponsored by food and beverage companies are exposed to sponsorship messages between 21,000 and 63,000 person-hours per week [20]. National and regional sporting organizations in Australia, with which community level sports clubs are often associated, were also found to be sponsored by unhealthy food companies [29].

In Canada, much like in other countries, there are large restaurant chains that have well-known sports sponsorship programs that have considerable reach [17,18,23]. Given that these same companies advertise heavily across various media channels, the effects of these sponsorship programs on consumer behavior may be greater due to the cumulative exposure to their brand and promotional messages. The extent to which Canadian children are exposed to food company sponsorship from both local businesses and large corporations while engaged in organized sports, has yet to be documented. Considering that three quarters of Canadians aged 5–19 years participate in organized sports [42], this form of marketing constitutes an important avenue by which food companies can target children. As such, the purpose of this pilot study was to determine the frequency and nature of food company sponsorship of children’s sports clubs in a Canadian context. Specifically, this research sought to answer the following questions: (1) What percentage of sports clubs are sponsored by food companies? (2) How frequent are food company sponsors relative to other sponsors? and (3) What type of food companies are sponsoring children’s sports clubs? It was hypothesized that food company sponsorship would be frequent, with fast food restaurants being the most common type of sponsor.

## 2. Materials and Methods

### 2.1. Sports Club Sampling

Using a national sports participation survey conducted in 2010, the five sports with the highest participation among Canadian children aged 5–14 years were selected for inclusion in this study [19]. These sports were soccer (42% participation), competitive swimming (24%), ice hockey (22%), basketball (16%), and baseball (14%) [43]. Sports clubs with members under 18 years were then identified in the established boundaries of Ottawa (Ontario), the national capital of Canada [44,45]. This city was selected because results could inform municipal food marketing restrictions currently being considered by the city’s local council [46].

Children’s sports clubs in Ottawa were identified and surveyed as per the methodology used by Maher et al. [22]. First, all national and regional sporting association websites were first identified using Google with the keywords, “association”, the sport name, and “Canada”, “Ontario” (the province where Ottawa is located) or “Eastern Ontario”. Identified sporting association websites were then examined for lists of sports clubs located in Ottawa. Ottawa sports clubs were also identified with Google searches using the key words “Ottawa”, “club”, and the sport name. To be included in the study, sports clubs had to satisfy the following inclusion criteria: (1) have members under 18 years old; (2) have their own website; 3) have their own club name; and 4) have information about their club available for website visitors [22]. Sports clubs that did not satisfy these criteria or that had inaccessible weblinks were excluded from the study (N = 27).

### 2.2. Data Collection

Sports club websites were surveyed for evidence of sponsorship by a trained undergraduate student (O.E.) between September and December 2018. All pages of club websites were examined including any publications (e.g., newsletters, reports). If a search field was available on club websites, “sponsorship” was also searched. A company or organization was identified as a club sponsor if it was listed as an official sponsor or partner, if its logo/name appeared on the club website, if it sponsored a specific tournament or club event, if it was involved in a club fundraiser, and/or if it provided discounts, coupons, or other prizes to club member and families [21,22]. The name of both commercial (e.g., companies) and noncommercial (e.g., city counselor) sponsors were recorded. Regional or national sporting organizations with whom clubs were affiliated were not considered sponsors and were not recorded.

### 2.3. Classification of Sponsors

Sponsors were first classified as either commercial or noncommercial sponsors. Commercial sponsors were further categorized by sector including food, sports-related goods, services, and retailers, construction/renovation companies, financial services, health-related services (e.g., dental clinics, pharmacies), automobile-related retailers and services, among others (see Supplementary Table S1 for full list). Food company sponsors were further classified by food category including: fast food restaurants (i.e., restaurants where food is ordered at a counter), sit-down restaurants (i.e., restaurants involving table service with a menu), savory snacks (i.e., chips), supermarkets, sweet baked goods (i.e., retail bakery selling cakes), coffee, alcohol (i.e., microbrewery), and water. Fast food and sit-down restaurants were further categorized by type of restaurant (i.e., grills/sports bars/pub restaurants, diner, fast food pizza restaurant, rotisserie chicken restaurant, fast food burger restaurant, fast food cafe restaurant, fast food sandwich and/or salad restaurant, and restaurants serving ethnic foods). A description of the foods sold by each type of restaurant is provided in Supplementary Table S2. Restaurants were also classified based on the size of the restaurant chain as per Ontario’s 2015 Healthy Menu Act [47,48]. As such, restaurants were classified as a single restaurant (defined as having one food service location in Ontario), small chain restaurant (defined as having less than 20 locations), or large chain restaurant (defined as having 20 locations or more) [48].

### 2.4. Data Analysis

Descriptive analyses were conducted using IBM SPSS Statistics Version 26.0 (Armonk, NY, USA, IBM, 2018). Frequencies and medians were tabulated to describe the presence of sponsorship and food company sponsorship overall and by sport. The characteristics of food company sponsors were also described using frequencies for the overall sample and by sport.

## 3. Results

### 3.1. Sports Club Sponsorship

Sixty-seven sports clubs were identified in Ottawa with ice hockey accounting for the largest share of clubs (37%). The median number of sponsors per sports club was 3 and varied between 0 and 37 while the median number of food company sponsors was 0 and varied between 0 and 5 (data not shown). Commercial sponsors accounted for 95.2% of sponsors while noncommercial sponsors accounted for 4.8%. Sports-related goods, services, and retailers were the most frequent sponsors (25.3% of total sponsors), followed by food companies (16.3%), construction/renovation companies (10.6%), automobile-related retailers and services (8.0%), health-related services (6.4%), and banks and financial services (5.4%) (Table S1).

Overall, 73% of sports clubs received some form of sponsorship while 40% received sponsorship from a food and beverage company (Table 1). Food company sponsorship was most frequent among soccer clubs (62% of clubs), followed by ice hockey (44%) and competitive swimming (43%). One quarter of sports clubs (n = 17) were sponsored by a fast food restaurant while 19% were sponsored by a sit-down restaurant (n = 13; data not shown).

### 3.2. Characteristics of Food Company Sponsors

Overall, 312 sponsors were identified across all sports clubs. Of these, 16% were food company sponsors (Table 2). Soccer and basketball sports clubs had the highest proportions of food company sponsors (24% and 22% of their sponsors, respectively).

Of the food company sponsors identified (n = 51), 45% were fast food restaurants, 41% were sit-down restaurants, 4% were supermarkets, and 10% promoted other food categories (Table 3). Hockey had the highest number of fast food sponsors (n = 13; 57% of its food company sponsors).

Of the 44 sponsors identified as restaurants, more than half (61%) were large restaurant chains with more than 20 locations in Ontario while 25% and 14% were small chain restaurants or single restaurants, respectively (data not shown). The largest share of restaurants were grills/sports bars/pubs (n = 16; 36%), followed by a fast food cafe restaurant (n = 9; 21%), a fast food burger restaurant (n = 6; 14%), fast food sandwich or salad restaurants (n = 4; 9%), fast food pizza restaurants (n = 3; 7%), restaurants serving ethnic foods (n = 2; 4%; e.g., Italian restaurant, Lebanese shawarma), rotisserie chicken restaurants (n = 2; 4%), and diners (n = 2; 4%) (data not shown).

### 3.3. Most Frequent Food Company Sponsors

Overall, 31 different food companies were identified as sponsoring one or more sports clubs. Tim Hortons was the most frequently reported food company sponsor (sponsoring 13% of sports clubs; n = 9), followed by McDonalds (9% of clubs; n = 6), Boston Pizza (4%; n = 3), Broadway Bar and Grill (3%; n = 2), Milano Pizza (3%; n = 2), Pita Pit (3%; n = 2), and Swiss Chalet (3%; n = 2) (data not shown).

## 4. Discussion

This is the first study to evaluate children’s potential exposure to food company sponsorship in sports club settings in Canada. Overall, our study found that more than one third of children’s sports clubs in Ottawa were sponsored by a food company. Ice hockey and soccer sports clubs were found to rely more heavily on food company sponsorship than other sports clubs.

The predominance of large and medium chain restaurants among food company sponsors is concerning given that most foods sold in these establishments in Canada, including their menu items for children, have been found to contain excessive amounts of calories, fat, sodium, and/or added sugars [49,50,51]. Larger restaurant chains advertise more heavily in other media, therefore, sponsorship of children’s sports clubs likely increases children’s exposure to these brands and may have a greater impact on their food preferences. Notably, one in four sports clubs in Ottawa were identified as sponsored by fast food restaurants, with Tim Hortons and McDonalds being the most frequently reported food company sponsors. This finding is not surprising given that both companies have well-known corporate sponsorship programs in Canada [18,19]. Tim Hortons, for instance, sponsors 300,000 children aged 4–9 years engaged in various sports every year including 5 to 6-year-old children enrolled in the introductory hockey program [52,53]. These children are often referred to as “Tim Bits” after the iconic donut holes sold by Tim Hortons. McDonald’s, for its part, sponsors more than 50,000 child hockey players aged 9–10 years old [52]. While no Canadian research to date has examined the potential impact of these sponsorship programs, a recent phenomenological study on the food experiences of Canadian adolescents enrolled in recreational hockey revealed that Tim Hortons’ sponsorship of their childhood team had cultivated brand loyalty with one participant quoted as saying “Tim Horton’s supported me when I played hockey, so I’m gonna support them when I wanna get food.” [54]. This quote epitomizes the desired effect that food companies likely seek by sponsoring children’s sports.

While we did not conduct a nutritional assessment of foods sold by sponsoring companies or brands, food categories or restaurants that are generally known to be unhealthy (i.e., fast food pizza, burger and cafe restaurants, alcohol, sweet baked goods, savory snacks) made up 41% of food company sponsors. Similarly, research conducted in Australia on the sponsorship of children’s sports clubs found that half of sponsoring food companies were not deemed to be “healthy” [21]. Direct comparison with this study and others is difficult, however, as methods used to classify food company brands or sponsors to assess their healthfulness diverge and remain a challenge in both research and policy. In Australia, for instance, Kelly et al. established criteria for defining a “healthy” sponsor through a consensus approach involving ten experts from health-related fields [21]. Other research examining food company sponsorship have defined unhealthy sponsors as those where more than 50% of their products do not meet established nutrition criteria [27]. While the latter method could be considered more objective, collecting nutrition data for every item associated with a brand, particularly restaurants, is a very onerous endeavor. Furthermore, defining healthy and unhealthy sponsors in this way may be ineffective in practice. For instance, even though most products associated with a brand may be considered healthy, a company could spend a disproportionate amount of its marketing expenditures on promoting its unhealthy products. Criteria for defining unhealthy sponsors should, therefore, consider other factors such as marketing expenditures or sales data.

### 4.1. Policy Implications

In recent years, the Government of Canada considered passing a law that would have restricted the marketing of foods and beverages high in saturated fat, sugar, and sodium to children under the age of 13 years in various media channels and settings [55,56]. This law, however, would not have prohibited food company sponsorship of children’s sports clubs as this activity had been explicitly excluded from considered restrictions over concerns regarding its potential impact on the financial viability of sports clubs and the affordability of sports participation [52,57,58]. In Canada, these concerns may be warranted as CAD 495 million were spent on the sponsorship of amateur sports in 2017 [59]. The unique contribution from the food industry however is unknown. Research from Australia found that sponsorship, for most children’s sports clubs, accounted for less than a quarter of their revenue, making the contribution of food companies likely nominal [21]. Our findings also suggest that these financial concerns may be overstated. Of the 321 sponsors identified as supporting children’s sports clubs in Ottawa, only 16% were food related. Given the predominance of nonfood sponsors, it seems likely that sports clubs could find alternative sponsors if food company sponsorship was restricted. To fully understand the financial implications of sponsorship restrictions, more research in Canada is needed.

To advance policymaking and inform advocacy in this area in Canada, research is also needed to understand stakeholders’ perceptions of food company sponsorship as well as their support for its restriction. Despite the documented effects of sponsorship on the attitudes, perceptions, and behavioral intentions of adults and children alike [32,35,36,37,38,54], these effects may not be widely known or recognized among the broader public, including parents. For instance, an Australian study found that less than half (48%) of surveyed parents thought children under 15 years were influenced by the sponsorship of their community sports club [60]. Perception of company motives may also diverge considerably. For example, some parents view the food industry’s involvement in community activities, including the sponsorship of children’s sports, as deceptive and unethical while others value these initiatives and perceive them as an act of good will [61]. Despite these mixed perceptions, research in other countries have suggested broad (48–76%) support for restrictions to food company sponsorship of children’s sports and community events attended by children, with fast food and soft drink companies being identified as particularly inappropriate sponsors [60,62,63]. Such research is needed in Canada.

### 4.2. Strengths and Limitations

The limitations of this study are important to highlight. Since it only examined sports club sponsorship in Ottawa, results are not generalizable to the rest of Canada. Furthermore, sponsorship information collected from club websites may not have been updated or complete. In addition, as some sports clubs encourage teams or individuals to seek out their own sponsorship [64,65], it is unclear whether sponsors listed on club websites include both club- and team-level sponsors. As such, it is possible that the frequency of sponsorship may have been underestimated. Since most websites did not report the promotional activities or benefits gained in exchange for sponsorship, the extent to which children are exposed to food company products or branding in this setting remains unknown. Future studies should be conducted in other regions in Canada, should validate sponsorship information with sports club officials, and should query the promotional activities (e.g., branded jerseys, discount coupons) to which children are exposed as result of sponsorship. While some findings are presented by sport, statistical testing to examine differences by sport could not be conducted as subgroups were too small. The current study also did not conduct a nutritional analysis of food products associated with sponsoring brands or restaurants. However, the classification of sponsors by food category and type of restaurant allowed for some assessment regarding the healthfulness of foods being promoted by way of sponsorship. Despite these limitations, this pilot study is the first to quantitatively assess food company sponsorship of sporting clubs in Canada and provides evidence that this activity is frequent. This pilot study also provided the data needed to justify a much larger study on food company sponsorship in Canada, which is soon to be underway.

## 5. Conclusions

Research has shown that unhealthy food marketing negatively influences children, prompting them to develop poor eating habits that may ultimately lead to poor health outcomes such as nutrition-related noncommunicable diseases and obesity [12]. Our findings suggest that food company sponsorship of sports clubs constitutes an important source of exposure to food marketing among children, some of which promotes unhealthy food categories such as fast food. Policymakers in Canada and abroad should consider restricting the promotion of such food categories through the sponsorship of children’s sports clubs.

## Figures and Tables

**Table 1:** Number and percentage of children’s sports clubs in Ottawa (Canada) with sponsorship and food company sponsorship, overall and by sport.

	Soccer (N = 13) n (%)	Competitive Swimming (N = 7) n (%)	Ice Hockey (N = 25) n (%)	Basketball (N = 13) n (%)	Baseball (N = 9) n (%)	All Sports (N = 67) n (%)
Clubs with sponsorship	11 (85)	5 (71)	17 (68)	8 (61)	8 (89)	49 (73)
Clubs with food company sponsorship	8 (62)	3 (43)	11 (44)	2 (15)	3 (33)	27 (40)

**Table 2:** Total number of sponsors and percentage of food company sponsors, overall and by sport, among children’s sports clubs in Ottawa (Canada).

	Soccer n (%)	Competitive Swimming n (%)	Ice Hockey n (%)	Basketball n (%)	Baseball n (%)	All Sports n (%)
Number of sponsors	68	25	146	27	46	312
Number of food company sponsors	16 (24)	3 (12)	23 (16)	6 (22)	3 (7)	51 (16)

**Table 3:** Food categories promoted by way of food company sponsorship of children’s sports clubs in Ottawa (Canada), overall and by sport.

	Soccer (N = 16) n (%)	Competitive Swimming (N = 3) n (%)	Ice Hockey (N = 23) n (%)	Basketball (N = 6) n (%)	Baseball (N = 3) n (%)	All Sports (N = 51) n (%)
*Type of Food Company Sponsor*						
Fast food restaurant	7 (44)	2 (67)	13 (57)	0 (0)	1 (33)	23 (45)
Sit-down restaurant	8 (50)	1 (33)	9 (39)	2 (33)	1 (33)	21 (41)
Supermarket	0 (0)	0 (0)	1 (4)	1 (17)	0 (0)	2 (4)
Alcohol	0 (0)	0 (0)	0 (0)	1 (17)	0 (0)	1 (2)
Sweet baked goods	0 (0)	0 (0)	0 (0)	0 (0)	1 (33)	1 (2)
Coffee	0 (0)	0 (0)	0 (0)	1 (17)	0 (0)	1 (2)
Savory snacks	1 (6)	0 (0)	0 (0)	0 (0)	0 (0)	1 (2)
Water	0 (0)	0 (0)	0 (0)	1 (17)	0 (0)	1 (2)

## Supplementary Materials

The following are available online at https://www.mdpi.com/1660-4601/17/9/3023/s1, Table S1: Number of sponsors of children’s sports clubs in Ottawa (Canada) by sector, Table S2: Description of foods served by restaurant type.
