# Supplementary material for: The Extent and Nature of Food and Beverage Company Sponsorship of Children’s Sports Clubs in Canada: A Pilot Study
Source: Int J Environ Res Public Health. 2020 Apr 27;17(9):3023. doi: 10.3390/ijerph17093023 (PMC7246505; doi:10.3390/ijerph17093023)
Supplement: Supplementary file 1 [file ijerph-17-03023-s001.pdf]

Title: The extent and nature of food and beverage company sponsorship of children's sports clubs in Canada: A pilot study

Authors: E. Pauzé, O. Ekeh, M. Potvin Kent

Supplementary Table S1 Number of sponsors of children's sports clubs in Ottawa (Canada) by sector

Sector	n (%)
Sports-related goods, services and retailers	79 (25.3)
Food companies	51 (16.3)
Construction/renovation companies	33 (10.6)
Automobile-related retailers and services	25 (8.0)
Health-related services	20 (6.4)
Financial services	17 (5.4)
Other	18 (5.8)
Non-commercial sponsors	15 (4.8)
Business services	12 (3.8)
Insurance companies or brokers	10 (3.2)
Hardware stores	9 (2.9)
Cleaning and environmental services	6 (1.9)
Realtor and property management companies	6 (1.9)
Hospitality services (e.g. hotel, cruise companies)	5 (1.6)
Gambling	2 (0.6)
Supplement companies	2 (0.6)
Unknown	2 (0.6)

Title: The extent and nature of food and beverage company sponsorship of children's sports clubs in Canada: A pilot study

Authors: E. Pauzé, O. Ekeh, M. Potvin Kent

Supplementary Table S2 Description of foods served by restaurant type

Restaurant category	Type of foods served	Restaurant name
Grills/Sports Bars/Pub restaurants	Sit-down restaurants serving alcohol and a variety of dishes including burgers and sandwiches, pasta dishes, steak, ribs, salads, fish and chips, pizza, nachos, chicken wings and some ethnic dishes	Boston Pizza<br>Big Rig Kitchen and Brewery<br>Jack Astor's Bar and Grill<br>Lone Star Texas Grill<br>D'Arcy McGee's<br>Broadway Bar and Grill<br>Don Cherry's Sports Grill<br>Hurley's Grill<br>Chances R Restaurant<br>Swan on the Rideau<br>Tail Gators<br>KS on the Keys
Diners	Sit-down restaurants that serve breakfast and a variety of entrees (e.g. burgers and sandwiches, chicken strips, meat loaf)	Denny's<br>Steele's restaurant
Rotisserie chicken restaurants	Sit-down restaurants whose primary menu item is roasted chicken which is served in various dishes (soups, sandwiches, quarter/half chicken meals)	Swiss Chalet
Fast food pizza restaurants	Fast food restaurant whose primary menu item is pizza. Other sold food items may include appetizers such as bread sticks, chicken wings, onion rings and nachos and other meals such as sandwiches and poutine.	Milano's Pizza<br>Jo-Jo's Pizza
Fast food burger restaurant	Fast food restaurant whose primary menu items include beef burgers, chicken sandwiches or wraps, chicken nuggets, fries,	McDonald's

	frozen desserts, breakfast sandwiches	
Fast food café restaurant	Fast food restaurant whose primary menu items include coffee drinks, donuts and other sweet pastries, sandwiches made with deli and other processed meats, soups, breakfast sandwiches	Tim Hortons
Fast food sandwich and/or salad restaurant	Fast food restaurants whose primary menu items include customized sandwiches and/or salads and who offer a variety of vegetable toppings	Pita Pit<br>Freshii
Restaurants serving ethnic foods	Restaurants that exclusively or primarily sell ethic foods (e.g. Italian restaurant, Lebanese shawarma restaurant)	Victoria Trattoria<br>Prince Gourmet Orleans
